# Supratentorial ependymoma: a comprehensive review of molecular classification, management strategies, and clinical outcomes (Part II of pediatric ependymomas across compartments)

**DOI:** 10.1007/s11060-026-05818-7

**Published:** 2026-10-08

**Authors:** Frederic A. Vallejo, Meredith A. Monsour, Nathan S. Fredricks, Derek S. Tsang, George Koutsouras, Timothy A. Ritzmann, Kristian Aquilina, Vijay Ramaswamy, Michael C. Dewan

**Affiliations:** 1 Department of Neurological Surgery, Vanderbilt University Medical Center, Nashville, TN USA; 2 Division of Pediatric Neurosurgery, Monroe Carell Jr Children’s Hospital at Vanderbilt University Medical Center, Nashville, TN USA; 3 John Sealy School of Medicine, The University of Texas Medical Branch at Galveston, Galveston, TX USA; 4 Department of Neurosurgery, The University of Texas Medical Branch at Galveston, Galveston, TX USA; 5 Radiation Medicine Program, Princess Margaret Cancer Centre, University Health Network, Toronto, Canada; 6 Children’s Brain Tumour Research Centre, University of Nottingham, Nottingham, UK; 7 Great Ormond Street Hospital, London, UK; 8 Division of Haematology/Oncology, Hospital for Sick Children, Toronto, Canada

**Keywords:** Pediatric ependymoma, Pediatric supratentorial ependymoma, YAP1-fusion, ZFTA fusion

## Abstract

**Purpose:**

To review the clinical, molecular, and imaging landscape of pediatric supratentorial ependymoma (ST-EP) and outline current management principles guiding surgical and adjuvant treatment decisions.

**Methods:**

Synthesis of literature regarding biology, diagnosis, and management of ST-EP, focusing on recently emerging molecular subtypes.

**Results:**

ST-EP is an important and clinically significant intracranial malignancy comprising roughly 30–40% of all ependymoma cases. The 2021 WHO molecular classification stratified ST-EP into ZFTA-fusion, YAP1-fusion, and NEC subtypes, fundamentally altering our understanding of this pathology. ZFTA-fused tumors represent the majority of ST-EP, are seen in older patients and harbor a relatively worse prognosis while YAP1-fused tumors, though rarer, are enriched for younger children and carry a substantially more favorable prognosis. Management of very young children presents a unique therapeutic challenge, balancing disease control against the neurocognitive sequelae of cranial irradiation and aggressive surgery in the developing brain.

**Conclusion:**

Across molecular subtypes, maximal safe surgical resection remains the dominant prognostic factor, with adjuvant focal radiotherapy well-supported based on tumor grade and extent of resection. Maintenance chemotherapy following radiotherapy has not demonstrated a survival benefit. Further multi-institutional investigation is needed to formalize molecularly-stratified treatment protocols, better characterize NEC subgroups, and identify targeted therapeutics for high-risk disease.

**Clinical trial number:**

Not applicable.

## Introduction

Pediatric brain and spinal cord tumors are the second most common childhood malignancy, accounting for approximately 20% of childhood cancers, with just under 5000 new cases annually in the United States. Despite this, they account for the greatest burden of morbidity and mortality among childhood cancers, reflecting an urgent therapeutic need. Ependymoma accounts for 6–12% of pediatric brain tumors, the third most common entity after pilocytic astrocytoma and medulloblastoma. Adult ependymoma often presents as a spinal lesion, whereas pediatric ependymoma is most commonly intracranial [1–6]. Pediatric ependymomas fall into two groups based on anatomic location: supratentorial and infratentorial. Supratentorial ependymoma (ST-EP) is less common, affects slightly older children, and comprises roughly 30–40% of cases, with several molecular subtypes [7]. 

Symptoms of ST-EP differ greatly based on anatomic location. For example, lesions may present with headache, seizures, sensorimotor and/or visual deficits depending on the neural pathways implicated. Patients with intraventricular extension, significant mass effect, or ventricular trapping may present with symptoms of obstructive hydrocephalus with increased lethargy. Current challenges in ependymoma management include timely diagnosis amid clinical symptom heterogeneity, extent of resection given lesion intimacy with critical neurovascular structures and eloquent tissue, and standardization of post-operative care across molecular subtypes: ST-ZFTA, ST-YAP1, NEC, and other non-canonical fusions. Clinicians also face risk-benefit discussions regarding observation (rather than adjuvant radiation) in completely-resected Grade 2 ST-EP or young children with YAP1-fused tumors, balanced against the risk of perioperative iatrogenic neurologic deficits following aggressive resection. Accurate diagnosis is crucial, as many tumors historically labelled supratentorial ependymoma are morphological mimics such as glioma, ETMR, astroblastoma, atypical teratoid rhabdoid, and embryonal tumors. Accurate diagnosis by identifying chromosome 11 fusions and/or methylation profiling is key to treatment decision-making.

While understanding the molecular drivers of these tumors is central to perioperative management, gross total surgical resection remains the most important modifiable prognostic factor, thus, disease management hinges on appropriate upfront surgical care. This review summarizes the current understanding of supratentorial pediatric ependymoma subtypes, discusses molecularly-tailored management strategies, and identifies knowledge gaps for future investigation.

## Molecular landscape

### Historical histopathologic classification

The WHO grading system for pediatric ependymoma has changed significantly over time. Despite sharing histologic hallmarks, such as monomorphic oval cells with “salt and pepper” chromatin and perivascular pseudorosettes, ependymomas differ significantly in response to therapy and in prognosis [8–10]. Grade 1 tumors were historically considered low-grade, including slow-growing subependymomas; Grade 2 (intermediate) tumors were labeled conventional or classic ependymoma, including myxopapillary ependymomas [6]; and Grade 3 tumors were described as high-grade, with anaplastic features and higher growth rates. This grading system’s reliability in clinical use is limited by high interobserver variance, even among experienced neuropathologists, and prognosis has not always correlated with grade [11]. 

Increased clinical access to DNA methylation, RNAseq, and gene fusion detection helped elucidate tumor molecular subtypes that may otherwise be histologically similar [12]. In 2015, molecular categories emerged for ependymoma, changing diagnostic paradigms across all compartments: supratentorial, infratentorial, and spinal [7]. Infratentorial and spinal ependymomas are discussed in parts 1 and 3 of this review; supratentorial ependymomas were stratified into *ST-ZFTA*, *ST‐YAP1*, and “not elsewhere classified.”

### 2021 WHO molecular entities

ST-EP are believed to originate from radial glial neural progenitor cells [13]. *ZFTA* fusion, formerly known as *RELA* ST-EP, remains the most common subtype, representing approximately 70% of cases, with *YAP1* fusion accounting for below 10%, and not elsewhere classified (NEC) tumors making up the remainder (Table 1) [9, 14]. *ZFTA* tumors typically affect slightly older children, with average age of 8, and have more rarely been observed in adults [15, 16]. The genome of posterior fossa and *YAP1* supratentorial tumors is much more stable than that of the *ZFTA* fusion tumors [17]. *ZFTA* (zinc finger translocation associated), formerly designated C11orf95, resides on chromosome 11q13 and fuses with *RELA*, causing constitutive nuclear translocation, contributing to DNA binding and transcription via zinc-finger domains [18, 19]. This fusion drives oncogenesis via a chimeric oncoprotein that upregulates NF- κB signaling [19]. The *ZFTA-RELA* fusion is the most common variant seen clinically, though other *ZFTA* fusions have been observed [20]. High levels of HDAC4 and SIRT1 mRNA expression were found to associate with significantly worse outcomes in ZFTA tumors, and drugs targeting HDAC4 and ABCG2 were predictive to be effective in in-silico models [21]. ZFTA-RELA fusion may be sustained in part by epigenetically modulated metabolic pathways involving itaconate to maintain oncogenicity [16]. Upregulated NF-κB pathways drive inflammation and proliferation. *L1CAM* is highly expressed on cell surfaces and is utilized as a surrogate for *ZFTA* fusion status, though this has not been reliably validated in prospective cohorts [22, 23].

**Table 1:** Summary of supratentorial ependymoma molecular subtypes

Molecular Subtype	Molecular Alteration	Typical Age	Anatomical Location	Imaging Characteristics	Prognosis	Current Therapeutic Implications
ZFTA-fused (ST-EPN-ZFTA)	ZFTA-RELA or ZFTA-MAML2 fusion, activating NF-κB signaling	Older children; wide age range	Cerebral cortex/parenchyma, often extra-ventricular; frontal lobe involvement common	Heterogeneous solid-cystic mass with eccentric cysts, internal calcifications, hemorrhagic foci, marked perilesional edema; restricted diffusion over solid component	Intermediate Higher-grade histology and more frequent recurrence. Homozygous CDKN2A/B deletion is an adverse marker	Maximal safe resection Adjuvant focal RT by grade and extent of resection. Chemotherapy has not demonstrated added benefit [14]
YAP1-fused (ST-EPN-YAP1)	YAP1-MAMLD1 or YAP1-FAM118B fusion; Hippo pathway dysregulation	Younger children, often infants (median age < 2 years)	Supratentorial, similar distribution to ZFTA-fused tumors	Proximity to ventricles, “garland” enhancement, data remain limited	Favorable in small series Recommend cautious interpretation pending larger studies	Surgery-first approach RT timing/deferral individualized in select young children. Observation is not yet standard practice [27, 28]
NEC No defining alteration	No currently identifiable defining molecular alteration on methylation profiling or fusion testing	Variable	Variable	No characteristic imaging signature established	Heterogeneous Depends on grade and extent of resection	Individualized management per grade/resection Molecular re-review recommended [65]
NEC Incompletely characterized / emerging entity	Distinct methylation or fusion signature present but not yet a recognized WHO entity	Variable	Variable	No characteristic imaging signature established	Heterogeneous Evolving as entities are further defined	Individualized management Consideration of re-profiling and clinical trial enrollment [66]

NEC = not elsewhere classified; RT = radiotherapy; PFS = progression-free survival. The NEC category is divided here into tumors with no currently identifiable defining molecular alteration and tumors representing incompletely characterized or emerging molecular entities distinct from ZFTA- and YAP1-fused ependymoma

NF-κB upregulation alone does not explain oncogenesis in *ZFTA* ST-EP, as CRISPR knockout and pharmacologic inhibition of NF-κB do not significantly affect tumor growth [24]. Rather, *ZFTA* fusion proteins interact with other transcription factor motifs, namely *PLAGL1* and *PLAGL2*, implicated in normal neuronal development [25]. Activation of downstream pathways such as MAPK, SHH, and SWI/SNF results in loss of differentiation, pushing cells toward a more anaplastic phenotype. *ZFTA* tumors also harbor secondary genomic alterations, most notably homozygous *CDKN2A* deletion, which inhibits RB and p53 function; homozygous but not hemizygous loss of *CDKN2A/B* was associated with poorer overall survival in the German HIT cohort, in contrast to findings from the Children’s Oncology Group ACNS0831 where homozygous loss of *CDKN2A/B* was absent from the favorable observation subgroup and was also found to not be prognostic [26]. This apparent discrepancy likely reflects differences in cohort selection and homozygous versus single-copy *CDKN2A/B* status across studies, and should not be read as evidence that *CDKN2A* status is uninformative in ST-EP absent from the favorable observation subgroup. Further evaluation of *CDKN2A* loss is required, particularly within the ongoing SIOP Ependymoma prospective trial (NCT02265770).

*YAP1* tumors are rarer than *ZFTA* fusion tumors, comprising less than 10% of ST-EP, with a median age under 2 years and a dramatically more favorable prognosis on standard-of-care treatment [7]. The largest *YAP1* study, published in 2019, demonstrated near-100% (one death during surgery) 5-year progression-free survival, with some patients not receiving any post-surgical treatment; these findings derive from a single, relatively small series, however, and should be interpreted with caution pending confirmation in larger cohorts [27, 28]. Because these tumors are rarer, naturally, there is a relative paucity of data available compared to *ZFTA* tumors. At the molecular level, *YAP1* ependymomas are primarily driven by gene fusions, most commonly the *YAP1-MAMLD1* fusion [28]. *YAP1* is a primary downstream effector of the Hippo tumor-suppressor pathway; fused with *MAMLD1*, the resulting protein becomes a powerful oncogenic driver [29]. 

A separate group of lesions, histologically similar to classic ependymomas but with distinct molecular profiles matching no well-classified subtype, is labeled “not elsewhere classified” (NEC). This category, representing roughly 20% of ST-EP cases, encompasses both tumors with no currently identifiable defining molecular alteration and tumors representing incompletely characterized or emerging molecular entities distinct from ZFTA- and YAP1-fused ependymoma [10]. This heterogeneous group may not respond to established treatment regimens for *ZFTA* or *YAP1* subtypes and represents a critical diagnostic and therapeutic challenge. These tumors require further molecular and clinical description to fully identify the heterogeneous molecular types that make up this class.

## Neurological imaging

ST-EP often present as large, heterogeneous masses, but recent studies suggest that *ZFTA-* and *YAP1-*fused tumors may have distinct features on imaging that can offer early hints at the underlying molecular characteristics [10, 30]. While these traits are no replacement for molecular analysis, they may help physicians guide management and understand the expected clinical course as each ependymoma subtype is more deeply understood.

### Features of ZFTA-fusion ependymomas

*ZFTA-*fusion ependymomas trend towards a few distinct radiologic signatures. These include preferential lesion location, morphology, enhancement on MRI, and common regions of recurrence. The *ZFTA* subtype is preferentially located in the cerebral cortex and parenchyma, often extra-ventricular, with frontal lobe involvement being most common [10]. Characteristically, these lesions extend from the pial surface to the ventricular margin. They are predominantly cystic, with large eccentric cystic components, internal calcifications, hemorrhagic foci, and marked perilesional edema [10, 31]. Most cases present with a heterogeneous solid-cystic appearance. On MRI, they exhibit heterogeneous contrast enhancement with regions of restricted diffusion over the lesion’s solid region. At recurrence, there is a tendency towards re-emergence along the dural flap [10]. Relative to *YAP1* and posterior fossa subtypes, supratentorial *ZFTA* subtypes less commonly involve the spinal cord (~ 12.5%) [32]. 

### Features of YAP1-fusion ependymomas

While *ZFTA* lesions tend towards the cortices and parenchyma, *YAP1* ependymomas are characteristically found intra- or periventricular [10]. In this location, these tumors are found with multi-nodular solid components with a classic “garland-shaped” pattern of solid enhancement on the periphery and diffusion restriction [28]. *YAP1* tumors may have peritumoral edema, but this is often less prominent than their *ZFTA* counterparts [10]. While these radiographic characteristics are early clues, they are not a replacement for molecular tumor analysis. As radiographic distinctions are sharpened by larger cohorts, these features and patterns may come into better focus and offer a clearer picture of what the multi-disciplinary team may expect for the patient’s treatment course.

## Surgical management

As with other pediatric ependymoma subtypes across craniospinal compartments, extent of resection (EOR) is the most important modifiable factor influencing overall survival, and gross total resection offers the best chance of cure [17, 33–37]. Alongside achieving a maximal safe resection, surgical goals include obtaining a tissue diagnosis, relief of mass effect, and treatment of hydrocephalus.

### Intraoperative surgical adjuncts and considerations

ST-EP can present anywhere within the supratentorial space and surgical approach should be tailored to lesion location. We utilize neuronavigation and ultrasound in nearly every case of ST-EP both to navigate relevant neurovascular anatomy and to assess extent of resection at various stages of the operation. Intraoperative MRI is also a valuable technique which has yielded higher rates of gross total resection in some series [38]. While awake craniotomies may facilitate speech and/or motor preservation, awake resections can be challenging or altogether impossible for the pediatric patients commonly affected by ependymoma [39]. For perirolandic lesions, intra-operative mapping and monitoring increase safety and optimize surgical resection.

Second-look surgery is an important aspect of surgical management in ependymoma and has led to improved long-term survival [40]. In a pan-Italian study, the overall survival of children who underwent second-look surgery prior to radiotherapy was similar to the larger group who underwent complete resection in the first procedure; morbidity was not significantly higher [41]. In this context it is appropriate to counsel families early that more than one surgical procedure may be necessary to obtain a complete resection. In another study, chemotherapy administered after the first surgery led to a significant reduction in tumor vascularity and facilitated second-look surgery with complete resection of residual disease [42]. Evaluation of post-surgical and pre-radiotherapy scans by a surgical advisory group has been helpful in determining which children will derive most benefit from second-look surgery [43]. 

**Fig. 1:**
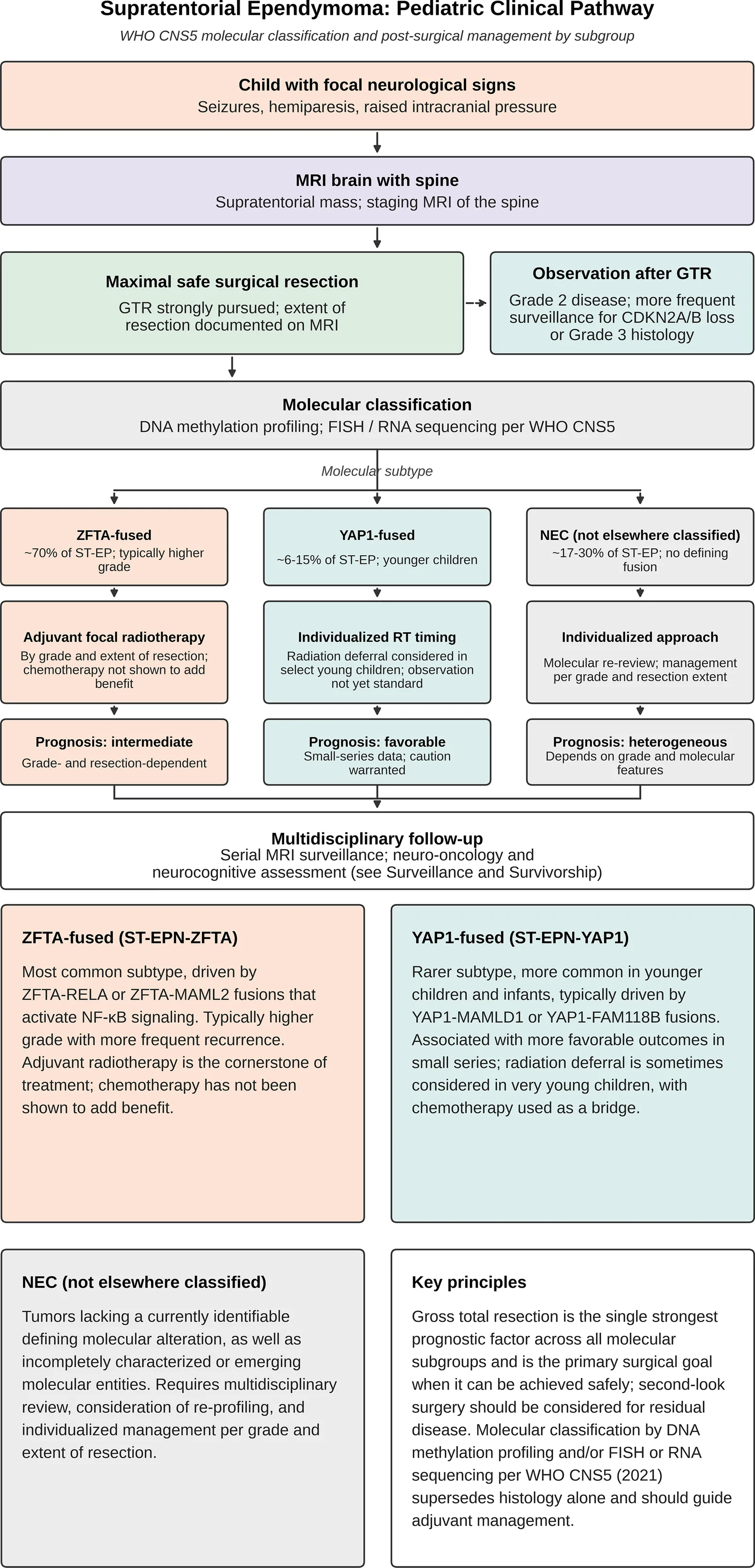
Supratentorial Ependymoma Management Pathway. EOR = extent of resection; GTR = gross total resection; NEC = not elsewhere classified; RT = radiotherapy; ST-EP = supratentorial ependymoma. Dashed arrow indicates an alternative pathway (observation) rather than a sequential step

**Fig. 2:**
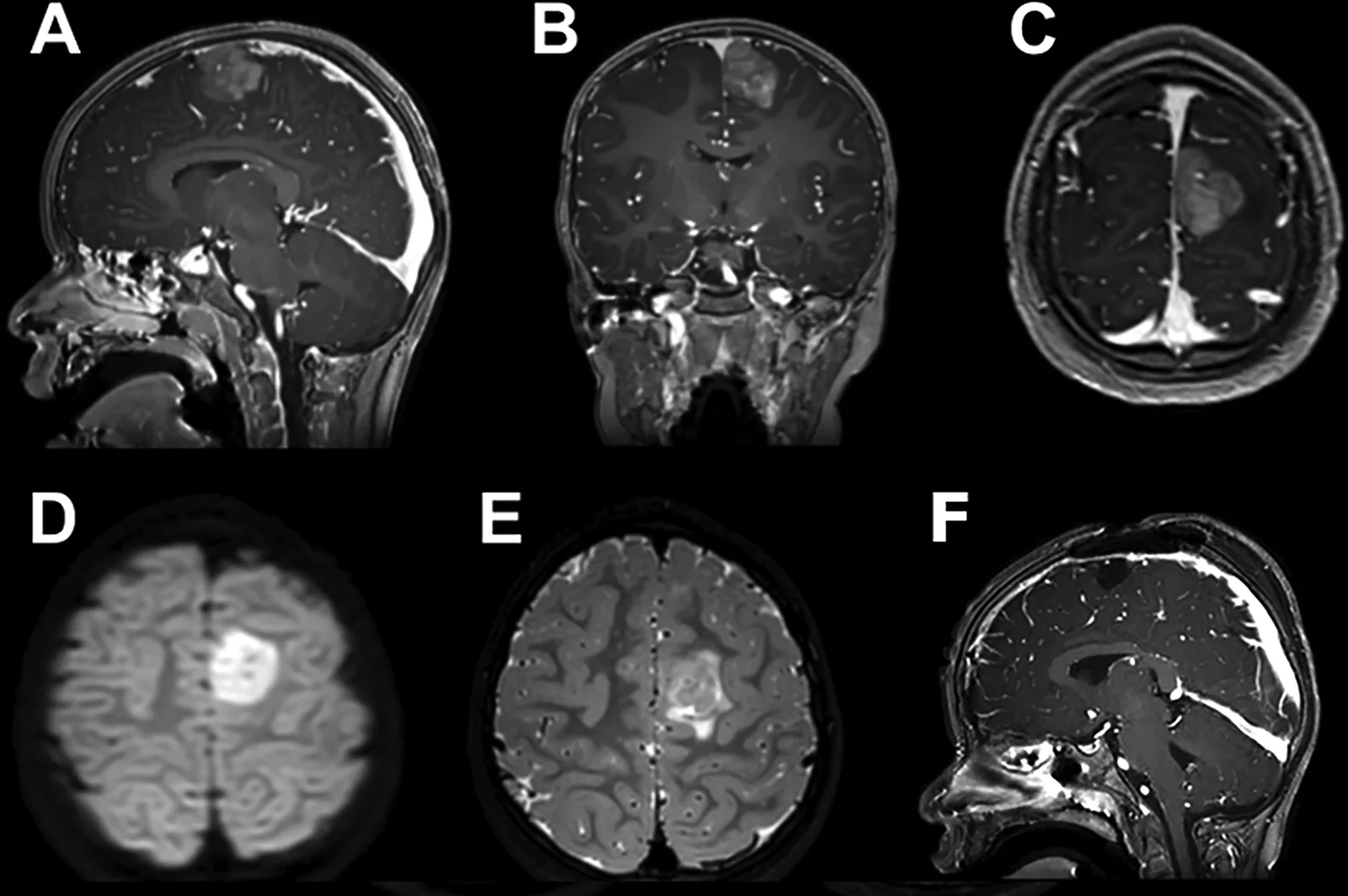
**A-F**: A case of ZFTA-RELA fusion. A 4-year-old male presented to the emergency department with several months of seizures with increasing frequency. MRI demonstrated a T2 hyperintense, heterogeneously enhancing 2.8 × 2.1 × 2.1 cm mass of the parasagittal posterior left frontal lobe. (**A**-**E**) The patient was clinically stable and was discharged home on dexamethasone with a plan for surgery later that week. In the operating room, he was positioned supine in Mayfield pins with the neck slightly flexed. Stealth navigation was registered, and a coronally oriented incision was planned just behind the coronal suture. A standard craniotomy was performed over the sagittal sinus, eccentric to the left. A gross total resection was successfully performed. (**F**) Pathology was consistent with ependymoma with WHO grade 3 histologic features, including brisk mitotic activity and microvascular proliferation. Histopathology sections showed densely packed cells with ovoid nuclei and brisk mitotic figures, scattered microcalcifications, and foci of perivascular pseudorosette formation. Molecular testing demonstrated a ZFTA-RELA fusion; no CDKN2A/B deletion was detected. No neurologic deficits were present post-operatively and he was discharged home following an unremarkable hospital course on post-operative day 2. After discharge, he completed adjuvant focal radiation (54 Gy in 30 fractions). (**A**, **B**, **C**) Pre-operative MRI showing a left parasagittal, heterogeneously enhancing lesion abutting the superior sagittal sinus. T1 post-contrast sequences., (**D**) DWI sequence with lesion restricting diffusion., (**E**) T2 sequence., (**F**) Post-operative MRI showing gross total resection and patent superior sagittal sinus

**Fig. 3:**
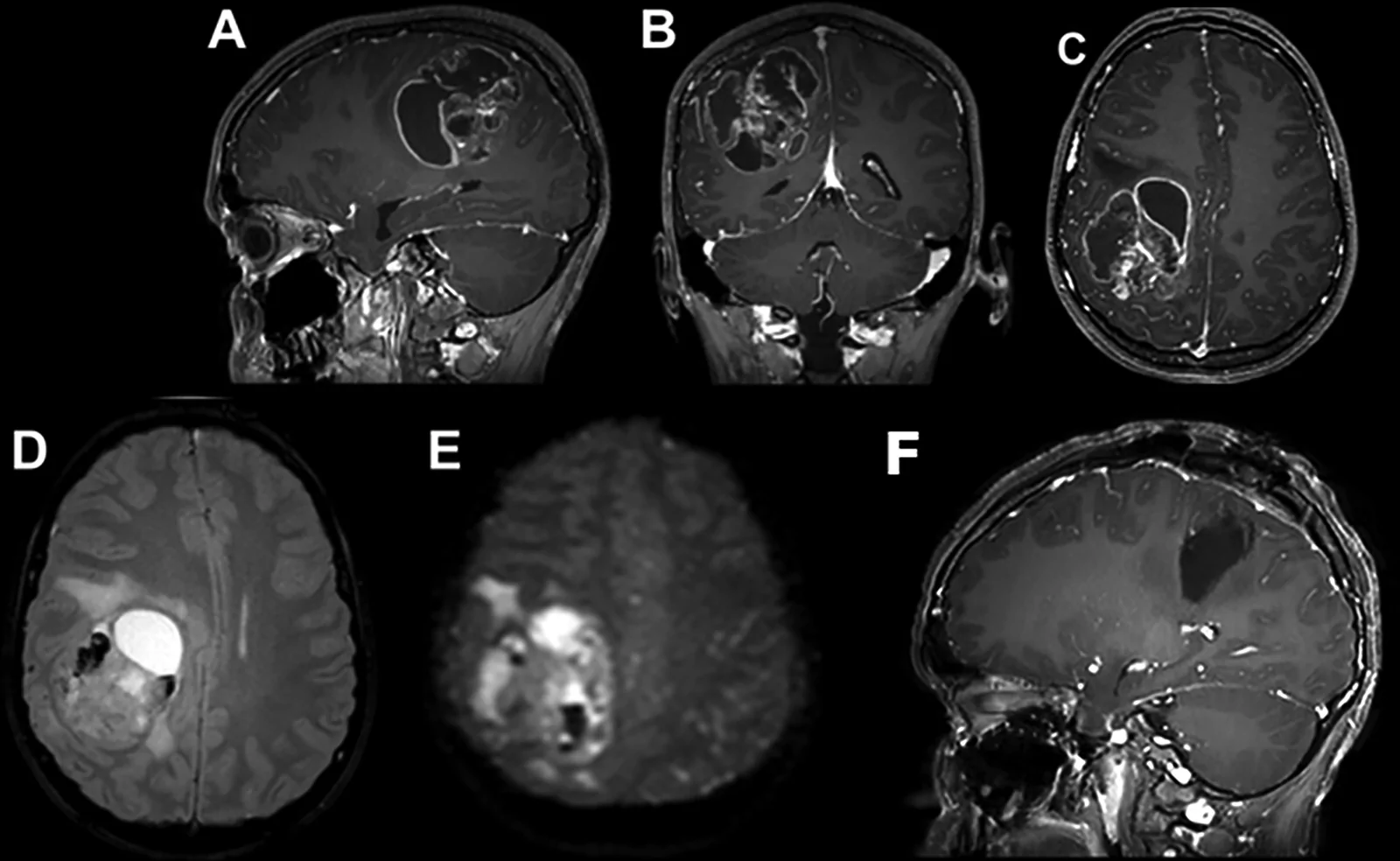
**A-F**: A case of ZFTA-RELA fusion with CDKN2A/B deletion. A 12-year-old female presented with a one-week history of headaches and nausea. MRI revealed a T1 and T2 isointense, heterogeneously enhancing 5.5 × 5.2 × 5.4 cm right frontoparietal mass with scattered cystic components, partial effacement of the left lateral ventricle, and 7 mm of leftward midline shift. (**A**-**E**) In the operating room, she was positioned supine in Mayfield pins with her head turned to the left and neck in gentle flexion. Stealth navigation was registered, and a curvilinear incision was planned extending laterally from just beyond the vertex to behind the right ear. A standard craniotomy was performed. Drainage of the large cystic component was achieved using an EVD catheter to minimize retraction on the brain. A gross total resection was successfully performed. (**F**) Pathology was consistent with an ependymoma with WHO grade 3 histologic features, including mitotic activity, microvascular proliferation, and necrosis. Histopathology sections showed cells with round-to-ovoid nuclei and scant eosinophilic cytoplasm arranged in sheets with perivascular pseudorosettes. Molecular testing demonstrated a ZFTA-RELA fusion; homozygous CDKN2A/B deletion was also detected. No neurologic deficits were present post-operatively, and she was discharged home following an unremarkable hospital course on post-operative day 2. After discharge, she completed adjuvant focal radiation (59.4 Gy in 33 fractions). (**A**, **B**, **C**) Pre-operative MRI showing right peri-rolandic heterogeneously enhancing lesion with cystic components and surrounding vasogenic edema. T1 post-contrast sequences., (**D**) T2 weighted sequence., (**E**) DWI diffusion restriction within the lesion., (**F**) Post-operative MRI showing gross total resection and reduction of peri-lesional mass effect and vasogenic edema

Once patients undergo primary resection and staging with CSF cytology and neuraxis imaging (MR spine with gadolinium contrast) at least 10–14 days post-operatively, physicians select adjuvant therapy appropriate for their patients’ clinical needs (Fig. 1). Modern treatment paradigms for *ZFTA* and *YAP1* are taking shape in this molecularly defined era; though specific guidelines by molecular stratification are not yet established, adjuvant regimens pre-dating these classes remain relevant guides for treatment decisions [44]. Two example cases from our institution are provided below (Figs. 2 and 3).

## Radiation therapy

Following maximal total resection, patients may begin RT via traditional modalities or proton beam, though protocols are not uniform: Ng. et al.’s cohort demonstrates use of E-HIT, COG, and institutional protocols for post-operative focal RT at the tumor bed [14]. Cumulative doses classically range from 54 to 59.4 Gy, adjusted to age and extent of resection (Table 2) [45, 46]. For localized disease, the standard adjuvant RT field is focally targeted at the tumor bed and does not include craniospinal irradiation, except with metastatic disease [32, 44]. No current guidelines exist in the literature for *YAP1* ependymomas; given the extremely young age of this population, observation after GTR is prudent but further prospective study is needed [28].

**Table 2:** Radiation and observation approach for localized supratentorial ependymoma, stratified by tumor grade, molecular subtype, CDKN2A/B status, and extent of resection

Tumor Grade	Molecular Subtype	CDKN2A/B Status	Extent of Resection	Recommended Approach	Evidence Level / Source
2	ZFTA-fused or NEC	Intact	GTR	Observation is a reasonable approach, with close surveillance; salvage surgery and focal RT reserved for recurrence	Established ACNS0121 (61% 5-yr EFS) ACNS0831 (66.9% 5-yr EFS)
2	Any	Any	STR	Focal RT, 59.4 Gy	Established ACNS0121 / ACNS0831
3	ZFTA-fused	Intact	GTR	Possible observation with increased surveillance frequency, or adjuvant focal RT prior to recurrence; requires individualized multidisciplinary discussion	Limited Not yet prospectively validated for Grade 3 disease
3	ZFTA-fused	Deleted (homozygous CDKN2A/B)	GTR	Adjuvant focal RT preferred over observation. 59.4 Gy if age ≥ 18 months; 54 Gy if age < 18 months	Limited Retrospective association with adverse prognosis (E-HIT-2000 cohort), not prospectively validated
3	Any	Any	STR	Focal RT: 59.4 Gy if age ≥ 18 months; 54 Gy if age < 18 months	Established
Any	YAP1-fused	Not applicable	GTR	Individualized RT timing/deferral considered in select young children; observation not yet standard practice	Limited Small case series only; caution warranted pending larger cohorts
Any	NEC	Variable / unknown	GTR or STR	Individualized approach; molecular re-review recommended	Limited Heterogeneous category with no defining molecular alteration

GTR = gross total resection; STR = subtotal resection; NEC = not elsewhere classified; RT = radiotherapy; EFS = event-free survival. Suggested radiotherapy doses are given in 1.8 Gy daily fractions

Suggested radiotherapy doses are given in 1.8 Gy daily fractions. Observation after gross total resection is supported by prospective cooperative-group data for Grade 2 disease, with 5-year event-free survival of 61% (ACNS0121) and 66.9% (ACNS0831) in this setting. This evidence does not yet extend to completely resected Grade 3 tumors or to YAP1-fused tumors of any grade, for which observation is not yet standard and should be considered only after individualized multidisciplinary discussion pending further prospective validation. More frequent surveillance is recommended for Grade 3 histology and/or homozygous CDKN2A/B deletion, given its association with adverse prognosis in the E-HIT-2000 cohort

Within the expanding molecular classification framework, ACNS0121 [46] evaluated 356 newly diagnosed ST-EP patients 1–21 years of age. Despite RELA fusion in 77% of supratentorial tumors, fusion status did not meaningfully alter survival among patients receiving prompt postoperative irradiation, questioning earlier assertions that RELA fusion-positive tumors carry a uniformly grim prognosis. The E-HIT-2000 trial [47], contributed molecular granularity, identifying homozygous CDKN2A deletion within ZFTA-fusion tumors as a particularly poor prognostic marker, conferring a 5-year PFS of 19% versus 81% in CDKN2A wild-type counterparts (*P* < 0.0001).

Within ACNS0121, 11 children with grossly totally resected supratentorial grade 2 ependymoma were eligible for observation rather than immediate post-operative RT [46]. This cohort (stratum 1) had a mediocre 5-year event-free survival of 61% but 5-year overall survival of 100%; despite recurrence in nearly half after omission of post-op RT, all were salvageable with repeat surgery and RT. This conservative approach was further evaluated in ACNS0831, which enrolled 449 children, of whom 166 had supratentorial tumors and 37 had grossly totally resected grade 2 ST-EP eligible for observation [45]. In this group, 5-year EFS was 66.9% with no deaths from disease recurrence, showing that grade 2 ST-EP has an excellent prognosis and is suitable for close observation after GTR. Notably, no ST-EP patient in the ACNS0831 observation arm harbored a CDKN2A deletion, suggesting that despite issues with histological grade as a marker, such tumors were not selected into this group, raising questions about observation alone in tumors harboring CDKN2A homozygous deletions.

**Fig. 4:**
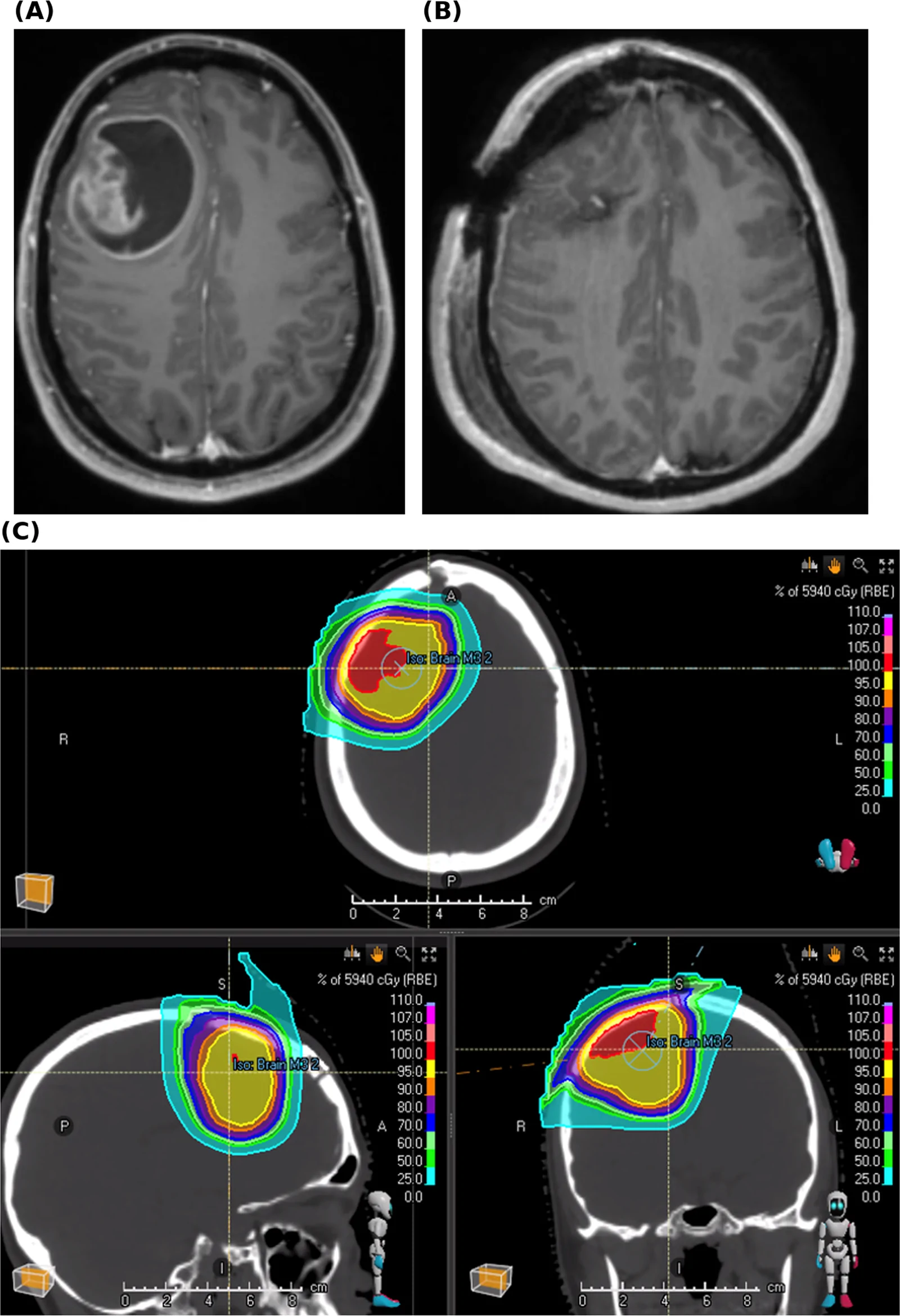
(**A**) Pre-operative MRI for a 26-year-old female who presented with a right frontal mass. (**B**) Post-operative MRI after gross total resection. Pathological evaluation revealed a supratentorial ependymoma, WHO grade 3, ZFTA fusion positive. (**C**) Proton beam dose distribution. This individual was focally treated to 59.4 Gy Equivalent in 33 fractions. The conformal nature of proton beam therapy permits the right frontal tumor bed to receive the prescription radiation dose, while the remainder of the brain receives zero or near-zero radiation exposure

A framework for radiation treatment of ST-EP is proposed in Table 2. The clinical target volume (CTV) expansion on the tumor bed shrunk from 10 mm in older studies [46] to 5 mm on ACNS0831 with excellent outcomes [45]; thus, 5 mm is the standard CTV margin. Because dose to uninvolved brain structures is associated with post-treatment cognitive detriment [48], proton therapy is recommended when available to reduce long-term toxicity while maintaining excellent tumor control [49]. An example of proton distribution for a patient with a grade 3 ST-EP is presented in Fig. 4.

The ACNS0121 and ACNS0831 studies show that a subset of ST-EP with complete resections, defined by intraoperative report and postoperative imaging, can be managed with observation alone. Although progression free survival is around 70%, nearly 100% of these patients can be salvaged with re-operation and radiotherapy at relapse; within ACNS0831, most patients were ZFTA-fused rather than YAP1-fused. Further studies are required to delineate whether ZFTA-fused tumors labelled histologically as “Grade 3” can also be managed this way.

## Chemotherapy

### Post-radiation chemotherapy

Across ST-EP, the benefits of chemotherapy after adequate resection have not been shown to improve survival. The ACNS0831 cohort of 449 patients compared outcomes between those randomized to RT alone and those randomized to maintenance chemotherapy after RT, showing no statistically significant difference in 5-year EFS (63.7% vs. 69.3%, *P* = 0.299) or 5-year OS (86.9% vs. 88.3%, *P* = 0.172) [45]. Chemotherapy seemed to be of no benefit in either supratentorial or infratentorial tumors. Extent of resection again emerged as the dominant prognostic variable: patients with subtotal resection fared considerably worse irrespective of chemotherapy exposure, while those with supratentorial grade 2 tumors achieving GTR had a remarkable 5-year OS of 100% [45]. The multicenter retrospective analysis by Ng et al. found that their cohort of 108 *ZFTA*-specific lesions had no survival benefit when treated with chemotherapy [14]. The European SIOP Ependymoma II study is due to report soon, following completion of recruitment; this large prospective aims to assess the impact of post-radiotherapy maintenance chemotherapy in children with completely resected ependymoma, and will provide important confirmatory data on this critical question [43]. The SIOP Ependymoma I/II protocol draws attention to the persistent therapeutic dilemma surrounding RT in very young children, with the UK maintaining more restrictive eligibility thresholds driven by concern that irradiation of the developing supratentorial brain carries substantial neurocognitive risk, even when proton beam is employed.

### Chemotherapy in young children

Several studies through the 1980s and 1990s evaluated chemotherapy alone after surgery as part of various baby brain protocols [43, 50–52]. While some, such as the Headstart protocol, suggested a potential benefit, the excellent outcomes seen in most YAP1 fused tumors and high progression-free survival with observation alone in completely resected ZFTA tumors in ACNS0831 call into question whether chemotherapy was ever necessary in this group. In children under 1 year, management is highly heterogeneous: many neuro-oncologists bridge with chemotherapy until an appropriate radiation age, though data supporting this practice remain sparse.

## Recurrent disease

### Recurrence patterns and molecular trends

Roughly half of children with intracranial ependymoma experience disease relapse [53]. Following initial intracranial ependymoma relapse, Tsai et al. found 5- and 10-year OS were 45.1% (95% CI 26.2–62.3) and 40.1% (95% CI 21.5–58.0) [54]. With each relapse, a further ~ 50% go on to subsequent relapse and for some children, ependymoma becomes a chronically relapsing disease [53, 55]. In posterior fossa A ependymoma there is clear evidence of molecular progression with the development of high-risk features at recurrence, though this has not been investigated in supratentorial disease and remains an area for investigation [56]. While less common in supratentorial lesions, these tumors may recur and require appropriate salvage management. Ependymomas often relapse locally, at the tumor bed of the primary lesion, though approximately 20–30% relapse metastatically across published series [32, 54, 57, 58]. When analyzed by molecular subtype, the literature shows that *ZFTA* tumors trend towards relapse at the initial tumor bed 66.7% of the time, with distant or combined distant/local relapse 33.3% of the time, with intracranial leptomeningeal dissemination observed more frequently in ST-EP and drop metastases to the spine occurring more frequently in PF-EP [59]. Distant relapse mostly occurred within the cranial space, often supratentorial, though infratentorial and meningeal spread to the spinal cord has been observed. The time to first recurrence for *ZFTA* tumors is approximately 2.5 years, though first relapse may occur 15 years or more after initial diagnosis [32]. *YAP1* ependymomas are known to relapse primarily at the initial tumor bed, though the record of recurrence remains limited due to the rarity of *YAP1-*based ependymomas.

Salvage treatment combines re-operation and repeat radiotherapy at the site of recurrence, with repeat resection being the driving prognostic therapy [53]. Chemotherapy has been employed at relapse, but its clinical benefit is unclear and no established guidelines exist for salvage treatment by subtype [57, 58]. Current literature highlights reoperation, when feasible, and RT as important steps in regaining control over recurrence [60]. Key factors for improved survival include GTR at reoperation and focal re-irradiation, which significantly improve OS and PFS at 5 years from relapse. Gross total resection of the recurrence prior to re-irradiation, and the use of craniospinal versus focal re-irradiation for local relapse, have each been associated with improved outcomes; conversely, salvage chemotherapy has been associated with worse outcomes, underscoring the primacy of resection and re-irradiation over chemotherapy in the salvage setting [45, 58, 61]. GTR at the treatment of a patient’s primary lesion was also associated with improved outcomes at relapse, further underscoring the importance of maximal safe resection at both therapeutic outset and recurrence [57]. 

## Surveillance and survivorship

Long-term follow-up after treatment for ST-EP should include serial MRI surveillance, though no definitive imaging interval has been established [62, 63]. Follow-up is tailored to each individual and should occur many years after initial diagnosis. Survivors are also at risk for treatment-related late effects, including neurocognitive decline associated with radiation dose to uninvolved brain structures, which is a rationale for proton-beam and other highly conformal radiotherapy techniques discussed above. Ongoing multidisciplinary survivorship care, incorporating neurocognitive, endocrine, and neurologic assessments remains an important but understudied component of long-term management for children treated for ST-EP [64]. 

## Future directions

Our understanding of ST-EP has improved in recent years with the definition of ZFTA-fusion and YAP1 molecular subtypes. Maximal safe resection remains the cornerstone of treatment across all subtypes; however, post-surgical stratification to differing treatment regimens based on molecular interrogation remains to be formally protocolized. Further investigation is warranted to more comprehensively characterize YAP1 and NEC ST-EP. Given the rarity of this pathology, future projects should aim to standardize genomic interrogation of supratentorial lesions, stratify patients into distinct molecular subtypes, aim to exploit emerging implicated genomic and epigenomic biomarkers, and combine multi-institutional data to determine which treatment paradigms work best for each subtype.

## Data Availability

No datasets were generated or analysed during the current study.

## Declarations

### AI use declaration

Claude AI (Anthropic) was utilized for assistance with manuscript editing for grammar and organization as well as assistance with formatting Fig. 1.
